# Bridging the gap, how interprofessional collaboration can support emergency preparedness for children with disabilities and their families: an exploratory qualitative study

**DOI:** 10.1186/s12889-023-15580-4

**Published:** 2023-04-28

**Authors:** Shelby K. Flanagan, Julia J. Sterman, Joseph R. Merighi, Rachael Batty

**Affiliations:** 1 School of Social Work, University of Minnesota, Twin Cities, Saint Paul, USA; 2 School of Health and Social Care, Edinburgh Napier University, Edinburgh, UK; 3 Center for Allied Health Programs, University of Minnesota, Twin Cities, Minneapolis, USA

**Keywords:** Family-centered care, Emergency preparedness, Interprofessional collaboration, Disabilities, Children, Pediatrics

## Abstract

**Background:**

Children with disabilities and their families are at higher risk during emergencies and disasters, which is often attributed to the lack of disability inclusion in emergency response as well as disparities in preparedness. This disparity speaks to a need for emergency preparedness that centers children with disabilities and their families. The purpose of this study was to elicit the perspectives of health professionals (nurses, occupational therapists, social workers), disability advocates, and public safety personnel (e.g., fire fighters, police officers, emergency management administrators) on what would enable these types of professionals to support family-centered emergency preparedness for families who care for children with disabilities. One goal of this research is to provide recommendations for practice and policy to improve safety outcomes for children with disabilities and their families in emergency situations.

**Methods:**

This study consisted of 46 qualitative interviews with nurses, occupational therapists, social workers, public safety personnel, and advocacy organization representatives about their role in emergency preparedness for families of children with disabilities. Qualitative content analysis was used to identify themes from participants’ responses.

**Results:**

Participants expressed interest in family-centered emergency preparedness, and stated that greater awareness, more education and training, increased networking between professions, and institutional support would enable their involvement.

**Conclusions:**

These findings have implications for the importance of interprofessional collaboration in supporting family-centered emergency preparedness for families of children with disabilities. Stronger interprofessional networks would help overcome many of the barriers identified by participants, and advocacy groups appear to be well-positioned to bridge the gap between these professionals and their areas of expertise.

## Background

The idea that people with disabilities, and families that include children with disabilities, are at higher risk of harm in natural hazards and other emergencies is well-established in literature [1–3]. Children with disabilities and their families are at a higher risk of injury or death in emergency situations than families that do not include children with disabilities [4, 5]. Emergency situations tend to exacerbate existing inequities, such as those experienced by disability communities and others who have less resource access, making families of children with disabilities less likely to be able to prepare and respond in emergencies [6–8]. In addition, few emergency response systems and policies are inclusive of the needs of people with disabilities [9, 10].

The lack of support for families of children with disabilities in emergency situations is compounded by the fact that these families are also less prepared for emergencies than the general population [11, 12]. The United Nations Convention on the Rights of the Child (UNCRC) confirms that all children have a right to life, survival and development; as well as a full and decent life which means that governments are obliged to do everything they can to make sure that children are able to live and grow to their full potential [13]. Family emergency preparedness is necessary to protect the emotional and physical well-being of children with disabilities and their families in an emergency. Lack of support for family emergency preparedness is a violation of their rights protected under UNCRC that causes families of children with disabilities to be more likely than families of typically developing children to be injured and die in emergency situations [4, 5].

As the prevalence of natural disasters rises and nations around the world continue to respond to the COVID-19 pandemic, these equity issues have only become more pressing [14, 15]. Not only are people with disabilities at greater risk in emergencies, but disasters and other emergencies create more disabilities [2, 16]. This cyclical effect combined with ongoing global climate catastrophe means that emergency preparedness that is inclusive of disability communities is becoming increasingly salient. Additionally, the COVID-19 pandemic has shown how emergencies can have the greatest impact on people with disabilities or chronic health needs, and how emergency preparedness policies often exclude the needs of people with disabilities [2, 16].

Most research on family emergency preparedness has focused on individual family preparedness. For example, a number of education interventions designed to increase the preparedness of families that include children with disabilities have been tested and found to be effective in increasing preparedness as measured by a generalized checklist of emergency supplies [17–19]. However, self-reported preparedness measures may not be an accurate measure of how a family will fare in an emergency situation [20] as each family has unique needs, and families that include children with disabilities in particular may have specific needs that are not generally considered in emergency preparedness planning. Therefore, a general checklist of supplies and planning steps may be inadequate for the planning needs of families of children with disabilities and chronic health conditions, as they cannot take into account each family’s individual capabilities and support needs. Measuring intervention effectiveness through item checklists does not evaluate whether a family’s supplies indicate planning ahead for an emergency, or having items by chance, and excludes the dynamic nature of forming a plan.

Formal and informal support networks are important enablers of emergency preparedness in disability communities. Informal support networks, such as social support and community participation, were associated with preparedness among older adults [21], and informal social support was associated with preparedness in a study of adults with disabilities that assessed preparedness by asking specific questions about planning for various emergency scenarios [22]. Informal networks build community resilience, a community’s capacity to adapt and respond to events like emergencies and disasters, which is an important facet of emergency preparedness [5, 23]. The growing acknowledgment of the importance of support networks is reflected in a movement toward “whole community preparedness,” which emphasizes the involvement and inclusion of all community members in emergency preparedness [24]. Specifically, the whole community approach means understanding the needs of often-overlooked community members such as children with disabilities and their families, as well as considering all community members’ strengths in terms of how they can help build community resilience to emergencies [25, 26].

The whole community approach also advocates for formal networking, including collaboration between government agencies, members of non-profit organizations, health care professionals, public safety personnel, and other relevant professionals [24]. Previous studies have examined the role of occupational therapists [27], social workers [28] nurses [29, 30], public health and safety personnel [12, 31], and non-profit advocacy groups [24, 32] in emergency preparedness. For example, occupational therapists can help families of children with disabilities develop emergency preparedness skills such as evacuation planning and recognizing warning signs [33]. In addition, nurses can discuss preparing for emergencies with families, taking into account chronic health conditions that may affect their emergency plan [29]. Social workers can facilitate community resilience inclusive of families of children with disabilities by helping strengthen families’ connections to other members of their community and resources that may help them in an emergency [29]. Public health and safety professionals can incorporate the needs of people with disabilities into their emergency response planning, such as providing accessible communication [12]. Finally, advocacy organizations can connect families with resources that they need as well as support interprofessional collaboration [32, 34]. However, more literature is needed on the ways in which these professionals can work together and collaborate interprofessionally to support families.

Interprofessional collaboration generally means partnership and interdependence between different professional fields in which information, responsibilities, and goals are shared between members of each field [35]. In addition, interprofessionalism in health care not only emphasizes practitioners from different disciplines working together, but also those practitioners all working with the patient or family [36]. This type of collaboration is vital when facing complex health-related issues, such as emergency preparedness for families of children with disabilities.

Drawing on the frameworks of interprofessional collaboration and family-centered care for children with disabilities, we propose a model of family-centered emergency preparedness that highlights the need for networks between health, public safety, and advocacy organizations that place families of children with disabilities at the center, and are based around these families’ strengths, needs, and values.

### Purpose of the study

The aim of this qualitative study was to elicit the perspectives of health professionals (i.e., nurses, occupational therapists, social workers), disability advocates, and public safety personnel (e.g., fire fighters, police officers, emergency management administrators) on what would enable these types of professionals to support family-centered emergency preparedness for families of children with disabilities and chronic health conditions. Nurses, occupational therapists, and social workers were chosen as the population for this study because these health professionals often work directly with families, and are more likely to have the time to engage in conversations about emergency preparedness than other types of health professionals might. While emergency preparedness can refer to all types of emergencies, including events like war and pandemics, we specifically focused on preparedness for natural hazards (e.g., severe storms, floods, fires).

## Methods

### Study design

An exploratory qualitative research design was used to understand how health professionals who work with children who have disabilities, public safety personnel and advocacy organization representatives conceptualize family-centered emergency preparedness.

### Participants

Following approval by the University of Minnesota IRB, researchers contacted nurses, occupational therapists, and social workers who had indicated their interest in participating in an interview following completion of a survey about family-centered emergency preparedness. Snowball sampling was also used to recruit additional study participants in these three disciplines. In addition, researchers recruited disability advocates and public safety personnel through direct outreach to organizations. Advocacy organizations included entities that advocate for disability communities, such as autism, Down syndrome, epilepsy, and muscular dystrophy societies and associations. The selection of disability advocates and public safety personnel was done in a purposive manner to represent a variety of occupational roles, rural and urban localities, and organization types.

A total of 46 participants engaged in this study including 29 health care professionals: 9 occupational therapists (OT), 10 social workers (SW), and 10 nurses (N); 7 members of advocacy organizations (ADV); and 10 public safety personnel (PSP) (see Table 1). The advocacy group members represented organizations that work on behalf of people with specific disabilities such as epilepsy, autism, Down syndrome, and muscular dystrophy, as well as for children with disabilities in general. Of the public safety personnel, one was a police officer, one was a fire marshal, five worked in emergency management or community emergency preparedness, and three worked in emergency medical services. To qualify for inclusion in the study, participants had to work in Minnesota, have been in their role for at least six months, and speak English. Participants were recruited from the Minneapolis-Saint Paul metropolitan area as well as from greater Minnesota, to include urban and rural perspectives. Participants were asked to describe their gender identity and racial or ethnic identity: 73.9% identified as women and all but one participant described themselves as white, with one participant identifying as multiracial (see Table 1).

**Table 1:** Characteristics of the sample

	**Health care professionals**			**Public Safety Personnel (*n* = 10)**	**Advocacy Group Reps. (*n* = 7)**	**Total (*n* = 46)**
	**Social Workers (*n* = 10)**	**Occupational Therapists (*n* = 9)**	**Nurses (*n* = 10)**			
**Sample- specific description**	Practice-oriented professionals concerned with improving the welfare of children with disabilities and their families	Professionals who help children with disabilities and their families develop or recover the ability to do meaningful activities	Professionals who support health care needs for children with disabilities and their families	People who work in leadership positions in governmental departments of public safety and emergency planning	People who work in leadership positions in organizations that advocate for specific disability groups	
**Race**	90% white	100% white	100% white	100% white	100% white	98% white
	10% multiracial					2% multiracial
**Gender**	70% female	89% female	90% female	40% female	86% female	74% female
**Education Level**		22% Doctorate			(Not asked)	4% Doctorate
	90% Master	56% Master	20% Master	50% Master		46% Master’s
	10% Bachelor	22% Bachelor	70% Bachelor	20% Bachelor		26% Bachelor
			10% Associate	30% Associate		9% associate (15% not asked)
**Age (years)**	M = 43.5 SD = 12.6 Range: 27–63	M = 43.0 SD = 11.4 Range: 33–62	M = 32.9 SD = 11.3 Range: 24–62	M = 46.2 SD = 7.3 Range: 38–61	M = 42.3 SD = 13.8 Range: 26–64	M = 41.7 SD = 11.97 Range: 24–64
**Years of Experience in Current Role**	M = 19.7 SD = 11.4 Range: 6–38	M = 15.6 SD = 13.3 Range: 4–35	M = 10.5 SD = 11.8 Range: 2.5–42	M = 13.4 SD = 7.3 Range: 5–42	M = 7.0 SD = 6.5 Range: 2.5–19	M = 14.5 SD = 12.2 Range: 2.5–42

Table of demographic characteristics of all participants

### Data collection

#### Materials

Three distinct interview guides were developed for each participant group: health care professionals, public safety personnel, and disability advocates. These guides contained eight questions for nurses, occupational therapists, and social workers; seven questions for public safety personnel; and six questions for disability advocates. The questions were developed based on the second author’s review of the literature on emergency preparedness [12, 17, 37, 38]. Multiple iterations of the questions were prepared and discussed by the researchers prior to their use in the individual interviews. A final draft of each interview guide was reviewed and discussed with a content expert in emergency preparedness. During the interviews, participants were asked about their roles in family-centered preparedness for families of children with disabilities (see Table 2 for more details).

**Table 2:** Abbreviated interview questions

Participant Group	Health Professionals	Public Safety Personnel	Advocacy Organization Representatives
**Questions**	Tell me about your practice setting Probe: What population of clients/patients do you work with?	Tell me about some of your primary job areas related to emergency response and preparedness	Tell me about your advocacy role. Probe: If participant has a disability or chronic health condition, ask for a description
	From your perspective, what does family-centered care involve?	Tell me about what your organization does well with supporting community emergency preparedness	What does your organization do really well?
	“Stay Scenario”: Thinking of a family you’ve worked with– What if there was a severe snowstorm where this family lives and they had no power and couldn’t leave their homes for a week? ***Probe:*** What would they need to consider from the 8 capability areas for family preparedness?	When you think about emergencies, what are you worried about with families of children with disabilities or chronic health conditions?	If org doesn’t seem to support emergency preparedness: How could your organization include supporting or advocating for families of children with disabilities or chronic health conditions to prepare for emergencies?
	“Go Scenario”: What if there was severe flooding where this family lives and they had to evacuate to somewhere safe for a week? ***Probe:*** What would they need to consider from the 8 capability areas for family preparedness?	How could you embed support for families of children with disabilities and chronic health conditions into the work you are currently doing? ***Probe:*** Are there agencies you could partner with?	What does emergency preparedness support look like at your organization? ***Probe:*** How might your organization include more support or advocacy for emergency preparedness?
	Considering the stay and go scenarios above, if we could create a checklist of things that the family should do to prepare, what would that look like? Probes: What about a different family?	(About a version of the family-centered emergency preparedness resource for providers) What are important resources that we should include that we have not yet included?	I asked you many questions about preparing for emergencies. Is there anything we did not discuss that you think is important for us to know when it comes to helping families prepare for emergencies?
	Thinking back on our conversation on family-centered care, what might family-centered care look like in the context of emergency preparedness?	Imagine it is two years from now, and [your city, department] is supporting emergency preparedness for community members with disabilities and chronic health conditions in the best possible way. What does that look like? How did you get there?	Is there anyone else who would be important for us to talk to, to learn more about supporting families of children with disabilities to prepare for emergencies?
	Tell me about the last time you supported a client to engage in emergency preparedness ***Probes:*** What did that look like? How well did it work? How do you know? What would you do differently?	Is there anyone else who would be important for us to talk to, to learn more about supporting families of children with disabilities and chronic health conditions to prepare for emergencies?	
	What would engaging in family-centered emergency preparedness look like in your practice? Does your organization have a business plan to provide services in case of emergencies?		

Table of the interview questions for each participant group

#### Procedures

Two members of the research team conducted individual, semi-structured participant interviews via Zoom. Interviews lasted between 24 and 82 min (mean = 48 min) and were recorded and then transcribed verbatim.

### Data analysis

The research team employed qualitative content analysis as a method of deriving structure and meaning from the interview texts [39]. This consisted of a round of open coding with the completed transcripts, and generating a list of codes from the qualitative data. Next, the research team reviewed and discussed these codes, and merged codes into categories. Following this step, the researchers analyzed the categories through the lens of barriers and enablers of family-centered emergency preparedness, the topic of the original inquiry. Four themes emerged: Theme 1—Lack of awareness among health care professionals on the need for emergency preparedness and among public safety personnel on the needs of families who have children with disabilities; Theme 2—Lack of education and training on emergency preparedness and the needs of families who have children with disabilities; Theme 3—Need for more cross-sector collaboration and networking to address family-centered emergency preparedness; and Theme 4—Need for more time, funding, and institutional support for family-centered emergency preparedness.

## Results

This section provides a description of the four primary themes: lack of awareness, lack of education and training, need for more cross-sector collaboration and networking, and need for more time, funding and institutional support. Participant quotes are used to elucidate each of these themes based on the experiences and perspectives of the study participants. Table 3 identifies each of four themes with an exemplary quote, the number of participants who addressed the theme in their interview, and a recommendation based on our analysis of the theme.

**Table 3:** Themes

Theme	Sample Quote	Number of Participants Who Addressed Theme	Recommendation
Awareness	I don't know that interest would be an issue, because I think as soon as providers and bedside nurses become aware that this is a concern, they're more willing [to spend time discussing emergency preparedness with families]. I think it's more of an awareness issue than anything (N05)	3 N 3 OT 2 SW 6 PSP	Increased communication between the health professionals and public safety professions around emergency preparedness for families that include children with disabilities
Education and Training	I know I don't know a lot, I want to try to bridge that gap… because we can't just plan that everyone's an able-bodied person who can get out of their house, apartment… and drive, walk… on their own (PSP02)	2 N 1 SW 6 PSP	Health professionals and public safety personnel should share their expertise about emergency preparedness and children with disabilities, respectively, with each other
Networking and communication	[We work] on both sides, with families to understand those things, but then also, I've had a lot of opportunity to engage with EMS [emergency medical services], with police, with fire (ADV01)	8 N 7 OT 1 SW 8 PSP 7 ADV	Advocacy groups can facilitate networks between families, health professionals, and public safety personnel
Institutional support and funding	I don't think that there's really a structure on talking about emergency preparedness with families. I don't know that any [hospital where I’ve worked] ever had clear-cut procedure for how to prepare parents and families and caregivers for an event like this (N06)	5 N 1 OT 3 SW 3 PSP	Structural change in public safety to include children with disabilities, and in health care institutions to prioritize emergency preparedness

Table of the four main themes with example quotes

*N* Nurse, *OT* Occupational Therapist, *SW* Social Worker, *PSP* Public Safety Personnel, *ADV* Advocate

### Lack of awareness

Many health care professionals noted that there was a lack of awareness of the need for emergency preparedness in their professions, and similarly, many public safety personnel noted that there was a lack of awareness of the needs of children with disabilities in their field. Health care professionals expressed that they were not always aware that emergency preparedness is something they should discuss with families, and “a lot of it comes down to awareness of the need” (N05). One social worker stated that if families do not initiate these conversations, social workers will not likely do so: “if it's not an issue for families, it's not an issue for [social workers] either, which is the problem” (SW04). This indicates that, perhaps in an effort to be family-centered, social workers take their cues from families about topics of focus, but families do not always know to bring up emergency preparedness as an area of importance to be addressed with health care professionals. Interestingly, the health care professionals noted that they believed once their peers were aware of the need to address emergency preparedness with families, they would have interest in and ability to do so:

I don’t know that interest would be an issue, because I think as soon as providers and bedside nurses become aware that this is a concern, they’re more willing [to spend time discussing emergency preparedness with families]. I think it’s more of an awareness issue than anything (N05).

This statement indicates that an increase in awareness would have a noticeable effect on health care professionals’ engagement in family-centered emergency preparedness.

Similarly, public safety personnel noted that they lacked awareness of the specific needs of families that include children with disabilities or that this is a group they should be reaching out to in particular. One public safety specialist said that this topic had never come up in their experience in the field, and “that might be a gap… I can't say that we've done anything specifically with the special needs group” (PSP06). Public safety personnel overall expressed a lack of awareness of disability communities and their needs.

### Education and training

Another item that health care professionals and public safety personnel both noted was lacking in terms of emergency preparedness for families of children with disabilities was education and training. Many health professionals said that they have not received training on how to engage in emergency preparedness discussions, though they believe that they and their peers would be interested in such training. One nurse noted that there are nurse educators in their unit whose job is to provide training, but “they have to be given [emergency preparedness] information, which is where the barrier normally is” (N07).

Additionally, public safety personnel stated that they have noted a lack of training in their field on interacting with children or adults with disabilities. For example, one participant recounted when his commander asked him to provide training on interacting with autistic children, saying that he had “‘no understanding of autism and I know you have two boys on the spectrum. We need help.’” (PSP09). Participants noted that they would like more education on disability inclusion and enacting emergency preparedness with the families they see. For example, one public safety specialist in emergency management stated, “I know I don’t know a lot, I want to try to bridge that gap… because we can’t just plan that everyone’s an able-bodied person who can get out of their house, apartment… and drive, walk… on their own” (PSP02). It appears that knowledge and training in both sectors is currently insufficient, in that the health care professionals reported a lack of emergency preparedness training and the public safety personnel reported a lack of disability training. However, both groups endorsed interest in these topics and a desire for more knowledge.

### Collaboration and networking

Given the need for more awareness and education among health care professionals for emergency preparedness and among public safety personnel for disabilities, increased communication between these groups and families would likely support their capacity for family-centered emergency preparedness. Many health care professionals stated that an emergency preparedness conversation with a family should involve professionals from multiple areas, such as social work, mental health, nursing, and others. In addition, an occupational therapist noted that it would be helpful to have a “point person” (OT09) coordinate family-centered emergency preparedness through collaboration between sectors and linking families to necessary resources.

Public safety personnel expressed that they would like to improve their direct communication with families. One member of a fire department stated that “most fire departments, given the opportunity to connect with families, would really appreciate that” (PSP10). Another emphasized that it should be the responsibility of the professionals, rather than families, to reach out to disability groups rather than waiting for individuals with disabilities or their families to reach out. “So that's where we can help… the onus isn’t on the autism group to probably have that accomplished. I can make sure and facilitate that [the conversation] does occur” (PSP08).

Disability advocates expressed that they need to engage in networking to connect families of children with disabilities to emergency services. For example, one advocate reported:

[working] on both sides, with families to understand those things, but then also, I’ve had a lot of opportunity to engage with EMS [emergency medical services], with police, with fire. What we’re talking about is making sure that they have at least a general understanding of some of the unique challenges that can go along with ASD [autism spectrum disorder]… and truly this is another theme that transcends just autism. (ADV01)

Many disability advocate participants recalled situations in which they interacted with local emergency medical services and first responders about the needs of people with disabilities in their community. Advocacy organizations may be well-positioned to support improved communication between families, health professionals, and public safety personnel, as many advocacy group representatives described their primary role as connecting people with resources and organizations.

### Time, funding, and institutional support

To enable increased communication and training for disability and emergency preparedness content, participants expressed the need for more time, funding, and institutional support for family-centered emergency preparedness. Nurses and social workers expressed that time is “probably a barrier” (SW06) or even “the biggest barrier” (N05) to conducting emergency preparedness discussions with families, and that funding is always an issue when introducing new or revised practices into their work. Another nurse commented that having emergency preparedness conversations with families may not itself come with a great deal of time and funding constraints, but that training health care professionals to do that work may require more time and funding. “If we were going to actually prepare something for our whole pediatric population, I'm assuming that would be time… and they would need funding for [training and things]” (N09). Similarly, public safety personnel also expressed that funding may be a barrier for communication with and inclusivity of disability communities. For example, one participant said that “to do better [at disability inclusion] means more staffing around this and honestly money. It does take some funding and I really don’t have much of a budget to work with” (PSP05). Another participant, when discussing communication with disability communities, mentioned that sign language interpretation is “cost prohibitive” (PSP02). However, one public safety participant mentioned that often funding is available for training, potentially making disability inclusion training more feasible.

To create a framework for enacting emergency preparedness for families of children with disabilities, participants described a need for institutional change, such as within hospitals or public safety departments. One nurse said,

I don’t think that there’s really a structure on talking about emergency preparedness with families. I don’t know that any [hospital where I’ve worked] ever had clear-cut procedure for how to prepare parents and families and caregivers for an event like this (N06).

Another nurse also expressed that the institution may create barriers to introducing more emergency preparedness discussions. “Everything has to be approved by multiple higher ups before it can even get to my manager… which is a huge barrier in itself” (N07). This demonstrates that institutions may sometimes pose a barrier or simply lack a framework for family-centered emergency preparedness.

## Discussion

This novel study examined the role of health professionals, disability advocates, and public safety personnel in family-centered emergency preparedness for families of children with disabilities through the lens of these stakeholders’ perspectives and experiences. The findings suggest thatnurses, occupational therapists, and social workers often lack awareness of the need for emergency preparedness as well as education and training about facilitating emergency preparedness conversations. Public safety personnel, in comparison, tend to lack awareness of the needs of children with disabilities and their families, and require education and training about how to work with families that include children with disabilities. These participants’ experiences suggest that deficiencies in awareness could be resolved through increased networking and collaboration between health professionals and public safety personnel, and these networks could be supported and facilitated by advocacy organizations that are accustomed to working with all of the relevant stakeholders (health professionals, public safety personnel, and families that include children with disabilities). In addition, institutions such as local governments and health care systems could support the aforementioned awareness, education, and training.

Previous literature has established that support networks are an important aspect of emergency preparedness, and that health, public safety, and advocacy organizations all have a role in supporting emergency preparedness for families that include children with disabilities [5, 22, 23]. Health professionals and public safety personnel can facilitate or support family-centered emergency preparedness for families that include children with disabilities, but that they may not be aware of their specific roles in this effort [27–30]. Building on this knowledge, the present analysis demonstrates that there is a lack of intersection between those with expertise in disabilities (health professionals, disability advocacy groups) and those with expertise in emergency preparedness (public safety personnel). However, there are opportunities to bridge this gap. First, awareness can be raised among health professionals and advocacy groups of the need for emergency preparedness conversations with the families and communities they work with, as well as among public safety personnel and institutions of the presence and needs of people with disabilities in their constituencies. Many participants pointed out that these discussions could be incorporated into their existing practice, they just were not doing so yet, which may mean that awareness alone could make a significant change. In addition, there is a need for education and training among health professionals about emergency preparedness, and among public safety personnel about disability. More connections between these groups of professionals could facilitate such awareness and education.

Although facilitating communication between diverse groups and systems through cross-sector collaboration is necessary to solve complex social problems [34], it is one of the primary challenges in emergency preparedness [40]. Nonprofit organizations are possible facilitators of cross-sector collaboration [41]. The present analysis found that cross-sector collaboration through information sharing and networking between these groups, while currently minimal, could help overcome the knowledge gaps between them. Advocacy groups can, and in some cases already do, serve as a bridge between families, health care organizations, and public safety resources. This is consistent with the “whole community” approach to emergency preparedness, the existing literature about the role of these professionals in emergency preparedness, and the need for interconnectedness between their fields [24–26].

Growing awareness of emergency preparedness and increased connection between health professionals and public safety personnel could provide more opportunities for education and training.. Specifically, public safety personnel could teach health professionals how to discuss emergency preparedness with their clients, and health professionals could teach public safety personnel about the needs of children with disabilities and chronic health conditions and their families. Finally, interviews with health professionals, public safety personnel, and advocacy organization leaders highlighted how institutional support of family-centered emergency preparedness is necessary to provide the time, resources, and framework for interprofessional collaborative emergency preparedness discussions.

### Limitations

One major limitation of this study is that the sample of health professionals, public safety personnel, and advocacy organizations representatives was fairly homogenous in terms of race and gender. Though this is fairly reflective of the makeup of these professions in Minnesota, it still likely resulted in less consideration of the intersecting challenges that some families may face in diverse communities. In addition, the findings of this qualitative study only represent the perspectives of those included in the study– Minnesota-based professionals. There may be issues related to family-centered emergency preparedness that would be crucially important to professionals in other geographic areas that were not considered in our study. Finally, the issue of selection bias is relevant, as professionals who agreed to participate in a study about emergency preparedness for families of children with disabilities may be more likely to have interest in and motivation to discuss these topics than others in their field. Thus, individuals from the stakeholder groups who did not participate may know less about emergency preparedness (health professionals) and disability needs (public safety personnel).

### Implications and future directions

**Fig. 1:**
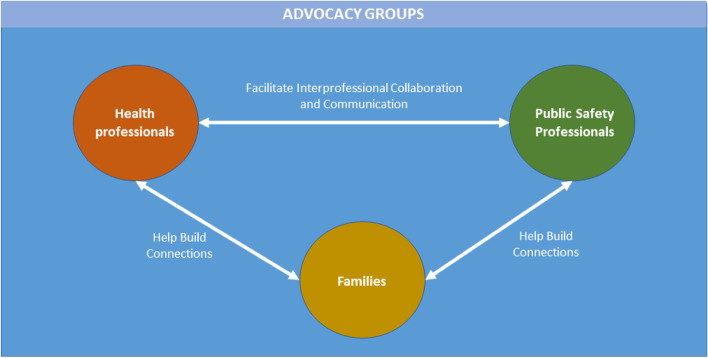
The Role of Advocacy Groups in Interprofessional Collaboration. A visual depiction of the role of advocacy groups in building connections and facilitating interprofessional collaboration and communication to support family-centered emergency preparedness for families of children with disabilities

The key themes identified in this study lend themselves to an increased focus on interprofessional collaboration in family-centered emergency preparedness for families of children with disabilities. Currently, many health professionals lack awareness of the need for emergency preparedness when working with families that include children of disabilities. Increased communication with public safety personnel would help resolve this lack of awareness, as emergency preparedness is an issue that many public safety personnel deal with every day. Similarly, many public safety personnel lack awareness of the needs of children with disabilities and their families, and increased communication with health professionals who work with these populations would provide opportunities for knowledge transfer. Health professionals need additional training and education on how to facilitate emergency preparedness discussions with their clients and patients, and public safety personnel need training and education on how to work with children with disabilities and their families. The sharing of information and coordinated decision-making and goal-setting that are key aspects of interprofessional collaboration [35] would also help fulfill these needs. Health professionals who work with children with disabilities and their families could train public safety personnel on the specific psychosocial and practical needs of this population, and public safety personnel with expertise in emergency preparedness could train health professional in facilitating conversations on emergency preparedness. This interprofessional collaboration could be supported by advocacy groups, which are well-positioned to do so by virtue of their experience networking with health professionals, public safety personnel, and children with disabilities and their families. Figure 1 provides a pictorial representation of the role of advocacy groups in supporting interprofessional collaboration and family-centered emergency preparedness. Advocacy groups can facilitate interprofessional collaboration and communication between health professionals and public safety personnel, as well as helping to build families’ connections with these groups.. Of course, families who care for children with disabilities likely have connections with health professionals, but advocacy groups can help strengthen those connections or expand them by helping families find additional resources and identifying community members who are not being served sufficiently by health professionals. Institutions prioritizing this collaboration and setting aside time and resources for the needed training and communication would also support implementation of family-centered emergency preparedness for children with disabilities and their families.

These findings have implications for practice in the fields of public health, emergency preparedness, and disability support. The findings point to a need for more cross-sector collaboration, in which those with expertise related to disabilities could train public safety personnel in interacting with and including children with disabilities and their families, such as by making communication accessible to children with disabilities and their families, interacting directly with these members of their communities, and understanding these families’ particular needs in emergency scenarios. Likewise, those with expertise in emergency preparedness can train health professionals in the emergency preparedness steps and strategies that they, in turn, can discuss with the families with whom they work. Advocacy groups and organizations could help support formal and informal networks to protect children with disabilities in disaster and emergency situations.

Currently, the onus is often on families to initiate emergency preparedness conversations with their health care providers, as well as introduce themselves to local emergency services. Within Minnesota and across the United States, this could be difficult or even impossible for families for myriad reasons including: immigration status preventing families from making themselves known to local authorities, experiences of racism in health care systems, or lack of insurance or limited access to care preventing families from discussing anything with health professionals that they view as nonessential. In addition, emergency preparedness may simply not be on a family’s radar, when more immediate concerns take precedent (e.g., food, shelter), and they may not be aware that these are conversations they should be having. Health professionals, disability advocates, and public safety personnel should take the lead in facilitating these conversations. This requires, as discussed above, a need for greater awareness and expertise in family-centered emergency preparedness and in the strengths and support needs of families that include children with disabilities.

Future research could explore the lack of knowledge of emergency-related family support needs. A consistent pattern in participants’ responses was health professionals not knowing or thinking about emergency preparedness as a need of families, or of public safety personnel not knowing about the needs of people with disabilities and their families. This points to the need for more inquiry into the specific support needs of families in contexts of emergencies, especially families that have children with disabilities, so that families’ needs can become better known by individuals who work in public safety and emergency preparedness. In addition, future studies need to explore how teachers and other school professionals can play an effective role in helping families be prepared for emergencies, especially families who care for children with disabilities and chronic health conditions.

## Conclusion

All children have a right to optimal health and safety, and children with disabilities deserve an equal chance to live and grow to their full potential. As climate change and a global pandemic make emergency preparedness an ever more pressing need [14, 15], family-centered emergency preparedness has increased importance. To our knowledge, this is the first study to ask nurses, occupational therapists, social workers, public safety personnel, and disability advocacy organization representatives about how they could better engage in family-centered emergency preparedness. Encouragingly, participants expressed that they would like to incorporate family-centered emergency preparedness into their professional routine and services, and that, in some cases, they can think of ways to do so. This indicates that awareness alone could result in a positive shift. To effectively enact whole community preparedness and create a more robust network to enable emergency preparedness for people with disabilities and their families, cross disciplinary collaboration should build bridges of communication and support between disability communities, health professionals, public safety personnel, and advocacy groups. This increased communication and intersection should help overcome many of the barriers to family-centered emergency preparedness that were identified by the participants in this study.

## Data Availability

The data from this study are not available publicly, as publication of full interview transcripts would violate participants’ privacy and the confidentiality agreement between participants and investigators, but are available from the corresponding author on reasonable request.
